# Functional Domain Analysis of the Remorin Protein LjSYMREM1 in *Lotus japonicus*

**DOI:** 10.1371/journal.pone.0030817

**Published:** 2012-01-23

**Authors:** Katalin Tóth, Thomas F. Stratil, Esben B. Madsen, Juanying Ye, Claudia Popp, Meritxell Antolín-Llovera, Christina Grossmann, Ole N. Jensen, Arthur Schüßler, Martin Parniske, Thomas Ott

**Affiliations:** 1 Department of Genetics, University of Munich, Martinsried, Germany; 2 Department of Genetics, Eötvös Loránd University, Budapest, Hungary; 3 Centre for Carbohydrate Recognition and Signalling, Department of Molecular Biology, Aarhus University, Aarhus C, Denmark; 4 Department of Biochemistry and Molecular Biology, University of Southern Denmark, Odense, Denmark; John Innes Centre, United Kingdom

## Abstract

In legumes rhizobial infection during root nodule symbiosis (RNS) is controlled by a conserved set of receptor proteins and downstream components. MtSYMREM1, a protein of the Remorin family in *Medicago truncatula*, was shown to interact with at least three receptor-like kinases (RLKs) that are essential for RNS. Remorins are comprised of a conserved C-terminal domain and a variable N-terminal region that defines the six different Remorin groups. While both N- and C-terminal regions of Remorins belonging to the same phylogenetic group are similar to each other throughout the plant kingdom, the N-terminal domains of legume-specific group 2 Remorins show exceptional high degrees of sequence divergence suggesting evolutionary specialization of this protein within this clade. We therefore identified and characterized the MtSYMREM1 ortholog from *Lotus japonicus* (LjSYMREM1), a model legume that forms determinate root nodules. Here, we resolved its spatio-temporal regulation and showed that over-expression of *LjSYMREM1* increases nodulation on transgenic roots. Using a structure-function approach we show that protein interactions including Remorin oligomerization are mainly mediated and stabilized by the Remorin C-terminal region with its coiled-coil domain while the RLK kinase domains transiently interact *in vivo* and phosphorylate a residue in the N-terminal region of the LjSYMREM1 protein *in vitro*. These data provide novel insights into the mechanism of this putative molecular scaffold protein and underline its importance during rhizobial infection.

## Introduction

Root nodule symbiosis (RNS) in legumes requires a complex molecular dialogue between the plant host and bacteria belonging to the *Rhizobiaceae* family. Upon perception of different flavonoid compounds released by the plant under nitrogen starvation, rhizobia secrete strain-specific lipochitooligosaccharide signaling molecules, called nod factors (NF), which are recognized by at least two receptor-like kinases (RLKs). In *L. japonicus* two LysM-type Nod Factor receptors NFR1 and NFR5 confer NF recognition specificity [1], [2], [3]. They trigger downstream physiological and morphological processes such as calcium-spiking, root-hair curling and activation of gene expression [4], [5]. In *Medicago truncatula* NFP and LYK3 have been described to serve these functions [6], [7], [8]. However, the fact that initial responses to NFs can be observed in an *hcl1*/*lyk3* mutant indicates the presence of another LysM RLK to be involved in NF perception. A closely related LYK4 protein has been proposed to be a likely candidate for a NF receptor component [8]. Phenotypical analysis of *M. truncatula* plants, where the NF receptors have been post-transcriptionally silenced by RNA interference (RNAi), and spatial analysis of receptor gene expression support the hypothesis, that these proteins are not only required for initial recognition of NFs prior to bacterial invasion but for the entire intracellular infection process. This was also suggested for the leucine-rich repeat RLK DMI2 from *M. truncatula* [9], [10]. While DMI2 and its homolog in *L. japonicus* SYMRK [11] have been originally isolated based on their infection phenotypes, recent genetic data suggest that SYMRK is rather required for nodule organogenesis and activation of a calcium-calmodulin dependent protein kinase (CCaMK) [12], a protein that decodes NF induced calcium-spiking.

Upon perception of NFs the root hair curls around rhizobia and entraps them in a micro-colony. Infection occurs via formation of infection threads (ITs), invaginations of the plant plasma membrane (PM) that surround rhizobia throughout the entire symbiotic interaction [13], [14]. While the IT progresses intracellularly towards the root cortex, cell divisions occur directly below the IT in outer cortical cells. They branch within the developing nodule primordium and finally release rhizobia into symbiosomes. These are spatially defined by the PM encapsulating the bacteria (the symbiosome membrane) and contain differentiated bacteroids, the nitrogen-fixing state of rhizobia.

We have recently shown that a Remorin protein from *M. truncatula* (MtSYMREM1) is able to interact with the putative NF receptors NFP and LYK3 as well as with DMI2. MtSYMREM1 localizes to infection threads within the nodular infection zone and symbiosomes membranes and is required for bacterial infection [15]. Remorins are plant-specific proteins that comprise a gene family with 16 members in *Arabidopsis thaliana* while only 10 genes have so far been identified in *M. truncatula* [16]. Members of all Remorin groups can be found in all higher plants, except group 2 Remorins, which are only present in legumes and poplar. This subgroup is comprised of two members. While MtSYMREM1 has so far only been described to be activated in response to rhizobia [15], the second gene is transcriptionally induced during arbuscular mycorrhiza symbiosis [17]. Furthermore recent data indicate that remorins belonging to the group 1 are function during plant-viral [18] and plant-microbe interactions [19]. While the exact mechanisms remain to be understood, the structural composition of Remorins with their highly conserved C-terminal region that harbors a coiled-coil domain and a set of conserved positively charged and aliphatic amino acid residues suggest similar core functions. In contrast, the N-terminal region is highly variable or absent in between the different Remorin groups [16] indicating functional specification.

## Results

### Evolutional divergence of *L. japonicus LjSYMREM1*

**Figure 1:**
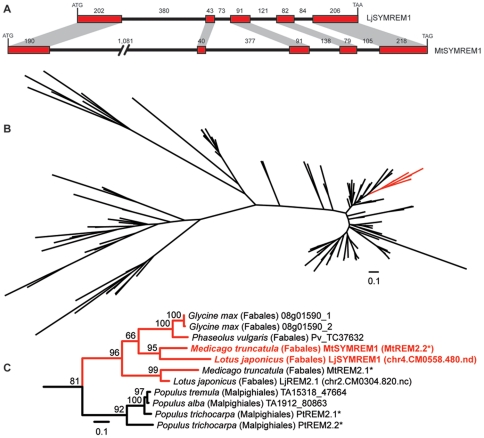
Identification and analysis of orthologous SYMREM1 genes and proteins. The *LjSYMREM1* sequence is similar to the previously published one of *MtSYMREM1* Both genes show the same exon-intron structure even though the *MtSYMREM1* gene is comprised of longer introns (A). Phylogenetic analysis based on 147 amino acid Remorin sequences using 101 unambiguously aligned residues in the conserved C-terminal region identifies the group 2 (B). Amino acid sequences of 11 group 2 Remorins from legumes and poplar were aligned and analyzed in 172 positions (C). MtSYMREM1 and LjSYMREM1 clearly cluster indicating that these proteins are orthologous to each other. Names marked with an asterisk were introduced in [16].

Legumes develop two main types of nodules. *Medicago truncatula* develops indeterminate nodules that have persistent meristem activity and are continuously infected. Other legumes such as *Lotus japonicus* develop determinate nodules that loose the ability to get infected and thus have a defined lifespan. Based on expression profiles [20], [21] we identified a *REMORIN* gene in *L. japonicus* that was significantly induced during nodulation, a feature that was also described for *MtSYMREM1* in *M. truncatula* [15]. The *LjSYMREM1* gene (chr4.CM0004.60.r2.d; http://www.kazusa.or.jp/lotus/) is comprised of 5 exons and 4 introns. Sequencing the genomic fragment of the putative *Medicago* ortholog *MtSYMREM1*, revealed a gene structure similar to *LjSYMREM1* (Figure 1A). Errors in the publically available annotation of *MtSYMREM1* (Medtr8g098650.1; http://www.medicagohapmap.org/) led to a previously reported incomplete annotation [15]. Thus the *MtSYMREM1* genomic sequence has been submitted to GenBank (Accession number JQ061257). Phylogenetic analysis revealed that *LjSYMREM1* and *MtSYMREM1* are closely related and directly evolved from a common ancestral gene, by speciation (Figure 1B–1C). They thus are orthologous genes.

Surprisingly, both proteins only share an overall similarity 67.1% (Table S1A) resulting from only 38.3% similarity in the N-terminal region while the C-terminal part of the protein is rather similar to MtSYMREM1 (85.3% similarity). Such low conservation was also found when comparing the N-terminal region of MtSYMREM1 with those of the closest homologs in soybean, poplar, common bean and grape wine (38.7% similarity) (Table S1A). This sequence divergence between the N-terminal regions of the SYMREM1 proteins of *Medicago* and *Lotus* is in sharp contrast to scores found for the symbiotic receptor-like kinases NFP/NFR5 and DMI2/SYMRK, the so-called ‘common *symbiosis*’ proteins DMI1/POLLUX, DMI3/CCAMK, IPD3/CYCLOPS, the putative transcription factors NSP2 and NIN and the late nodulin leghemoglobin 1 where the average sequence similarity is 81.9% with NIN only showing 67.5% similarity between the two legumes (Table S1B). This high sequence divergence of *Medicago* and *Lotus* SYMREM1 proteins that suggests high evolutionary pressure on the N-terminal region prompted us to functionally characterize the *Lotus* LjSYMREM1 protein, to analyze the contributions of the individual domains to SYMREM1 localization, function and to the interaction with the symbiotic RLKs NFR1, NFR5 and SYMRK.

### Overexpression of *LjSYMREM1* increases nodulation

**Figure 2:**
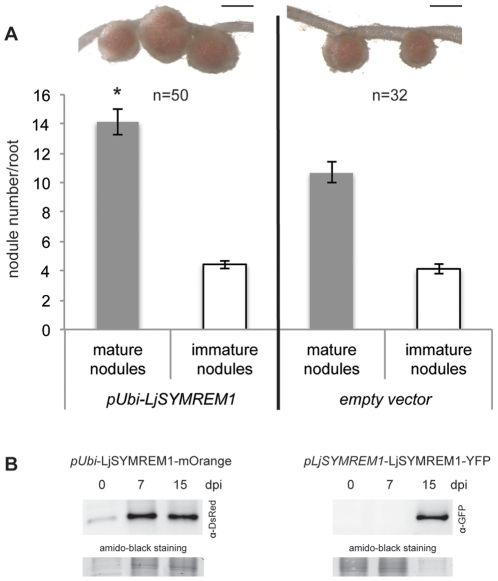
Overexpression of *LjSYMREM1* leads to increased nodule numbers. LjSYMREM1 was overexpressed as a mOrange fusion protein in transgenic *L. japonicus* roots (A). Nodule number and morphology was assessed 28 dpi with *M. loti* (MAFF303099-DsRed). Nodules were grouped into mature/pink and immature/white nodules and counted. Nodule morphology was not altered as indicated by the representative inlets above. Scale bars indicate 500 µm. Error bars represent standard errors and significance levels that were determined by student's t-test are indicated by an asterisk (p<0.01). Western Blot analysis to determine expression levels of *LjSYMREM1* in transgenic roots (B). Proteins from transgenic roots of chimeric plants expressing a *pUbi:LjSYMREM1:mOrange* construct (left) and stable transgenic plants expressing a *pLjSYMREM1:LjSYMREM1:YFP* construct (right) were transferred to a PVDF membrane and probed with the respective antibodies. Amounts of proteins loaded transferred the membrane is indicated by Amido black staining.

To show the importance of LjSYMREM1 genetically, we intensively screened the *L. japonicus* mutant population at RevGen, Norwich, UK (http://www.lotusjaponicus.org/tillingpages/homepage.htm) by a Targeted Induced Local Lesion in Genomes (TILLING) approach. Unfortunately, no potential homozygous knock-out mutant could be obtained while 15 non-allelic mutations that were identified with six being located in non-coding regions, four missense mutations, three silent mutations and one being located at the splice site did not show any phenotypical differences (data not shown). Thus we generated a LjSYMREM1:mOrange fusion construct that was driven by the *Lotus* poly-ubiquitin promoter (pUbi) [23] to assess the nodulation phenotype upon overexpression of *LjSYMREM1*. Transgenic roots expressing this construct were generated and inoculated for 28 days with *Mesorhizobium loti* (MAFF DsRed). Roots over-expressing *LjSYMREM1* developed significantly more mature nodules (24.6%; p<0.01) without any macroscopical alterations (Figure 2A) compared to the empty vector control while both genotypes exhibited similar numbers of immature nodules (bumps). However, transgenic roots overexpressing LjSYMREM1:mOrange did not show more infection threads at 28 dpi (neither mature nor aborted infection threads; data not shown).

To confirm overexpression of the construct we isolated proteins from transgenic roots expressing the *pUb:LjSYMREM1:mOrange* construct and showed presence of the fusion protein at different time points (Figure 2B, left panel). In contrast LjSYMREM1:YFP protein expressed in stable transgenic lines under control of the native promoter (described below) could only be detected in roots 15 days after inoculation with *M. loti* (Figure 2B, right panel). Expression of the transgene was also verified by microscopy prior to phenotypical analysis where patterns as described later in the text were observed. However, natively expressed LjSYMREM1 protein was never detected microscopically in root cells (see below).

### Spatial expression of the *LjSYMREM1* gene

**Figure 3:**
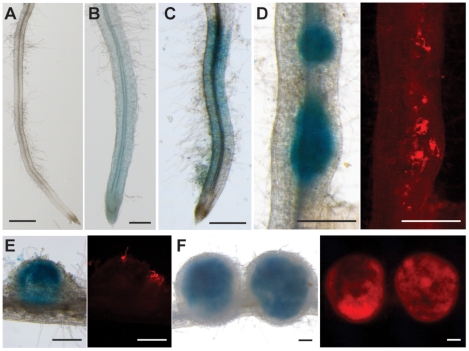
Analysis of *LjSYMREM1* promoter activity during rhizobial infection. Uninoculated transgenic roots transformed with the promoter:GUS construct (A). Application of 10^−8^M purified Nod Factors for 24 hours induced promoter activity in the root infection zone (1–5 cm above the root tip) (B). Root 48 hours after inoculation with DsRed expressing *M. loti* MAFF303099 (no infections) (C). Root segment with nodule primordia at 4dpi (D). Red fluorescence deriving from the bacteria shows their presence at the root surface. Young nodule at 6dpi with bacteria infecting the cortex (E). Mature nodules at 21dpi that are entirely infected by rhizobia (F). Bars indicate 500 µm.

Next we assessed spatio-temporal expression of *LjSYMREM1* since such data have not been provided for any *SYMREM1* gene. Thus we cloned a 975 bp fragment of the putative *LjSYMREM1* promoter (*pLjSYMREM1*) and generated transcriptional fusions to the β-glucuronidase (*GUS*) gene. The *_pLjSYMREM1_:GUS* reporter construct was transformed into *L. japonicus* roots using *Agrobacterium rhizogenes* mediated gene transfer. No GUS staining was observed in uninoculated transgenic roots after five hours of staining (Figure 3A). However, some weak staining of vascular tissue and root tips was occasionally observed after extended staining time, but this was also observed in roots transformed with the empty GUS-vector and was thus regarded as background staining (data not shown). To test promoter activation upon application of isolated NFs we applied 10^−8^ M isolated *Mesorhizobium loti* NFs as a droplet in the root hair elongation zone above the root tip to these transformed roots. This zone was previously described to be most susceptible to rhizobial infections [22]. GUS-activity was observed 24 hours post inoculation (hpi) in epidermal and cortical cells in the area where NFs were applied confirming inducibility of the *LjSYMREM1* gene by these bacterial signaling molecules (Figure 3B; S1A). Next we tested promoter activation during symbiotic interaction in transgenic roots carrying the *_pLjSYMREM1_:GUS* construct. Plants were inoculated with *M. loti* (expressing a fluorescent DsRed marker) by application of rhizobia to the whole root system and stained for GUS-activity 2, 4, 6 and 21 days post inoculation (dpi). As shown after NF application *LjSYMREM1* promoter activity was observed in a distinct zone above the root tip at 2 dpi (Figure 3C). Roots that had been inoculated for four days showed strong β-glucuronidase-activity that localized specifically around nodule primordia with progressing bacterial infection, while the epidermal staining, that was observed at pre-infection stages, was entirely diminished in these roots (Figure 3D). From 4 dpi onwards GUS-staining coincided with the presence of bacteria. In developing and mature nodules GUS-activity was detected in infected cells in the central zone of the nodule hosting nitrogen-fixing bacteroids but not in outer cortical cells (Figure 3E–3F). This was confirmed by sectioning these nodules prior to GUS staining. There, *LjSYMREM1* promoter activity was clearly found in inner nodule parenchyma cells that were not infected, as well as in infected cells (Figure S1B).

### Localization of LjSYMREM1 in legume nodules

**Figure 4:**
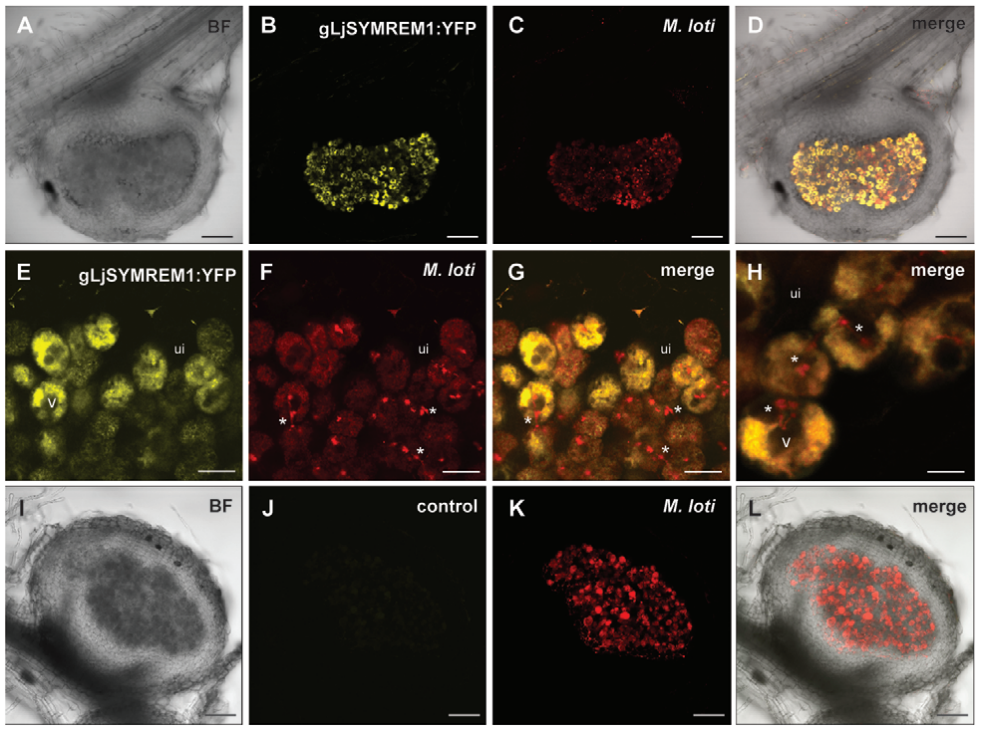
Stable transgenic *Lotus japonicus* plant expressing gLjSYMREM1:YFP. A genomic construct consisting of the *LjSYMREM1* native promoter (*_pLjSYMREM1_*) and the *LjSYMREM1* gene was fused to YFP (gLjSYMREM1:YFP). Roots were inoculated with *M. loti* MAFF303099-DsRed and three week old nodules of stable transgenic T2 plants were analyzed as 150 µm thick sections. Nodule of a transgenic *Lotus* plant shows strong YFP-fluorescence in infected cells that co-localizes with *M. loti* (A–D). Close-ups of infected (yellow) and uninfected (ui; no fluorescence) cells at the periphery of the nodule cortex (E–H). Vacuoles (V) are visible in the center of infected cells. Remnant trans-cellular infection threads lacking gLjSYMREM1:YFP protein are marked with an asterisk. Cross-section of an untransformed control nodule shows no fluorescence (I–L). Bars indicate 100 µm (A–D, I–L), 20 µm (E–G) and 10 µm (H). BF = bright-field.

To study localization of the native LjSYMREM1 protein, we generated a construct where the promoter together with the intron-containing version of *LjSYMREM1* that was amplified from genomic DNA was cloned and fused to the yellow fluorescent protein (YFP; gLjSYMREM1:YFP). Using *A. tumefaciens* mediated gene transfer we created stable transgenic lines in the *L. japonicus* ecotype MG-20 background. In T2 plants, we could not detect distinguishable YFP fluorescence in NF-treated roots due to high levels of intrinsic auto-fluorescence. However, a clear and specific YFP signal was detected in infected cells of mature nodules at 21 dpi (Figure 4A–4H). In comparison no YFP signal was detected in untransformed control nodules of MG-20 wild-type plants (21dpi) (Figure 4I–4L). In transgenic nodules the gLjSYMREM1:YFP fluorescence co-localized with the DsRed signal derived from *M. loti* expressing this fluorophore (Figure 4D,4G,4H and Figure S2A) suggesting localization of the protein on symbiosome membranes surrounding bacteroids in infected cells. A more detailed view on nodular infection threads also showed presence of LjSYMREM1 on these infection structures (Figure S2B). These data are in agreement with localizations reported for MtSYMREM1 that was detected by immuno-localization experiments on nodular ITs in the infection zone and on symbiosome membranes of indeterminate *Medicago* nodules [15].

### The C-terminal domain of LjSYMREM1 is mediating PM localization

**Figure 5:**
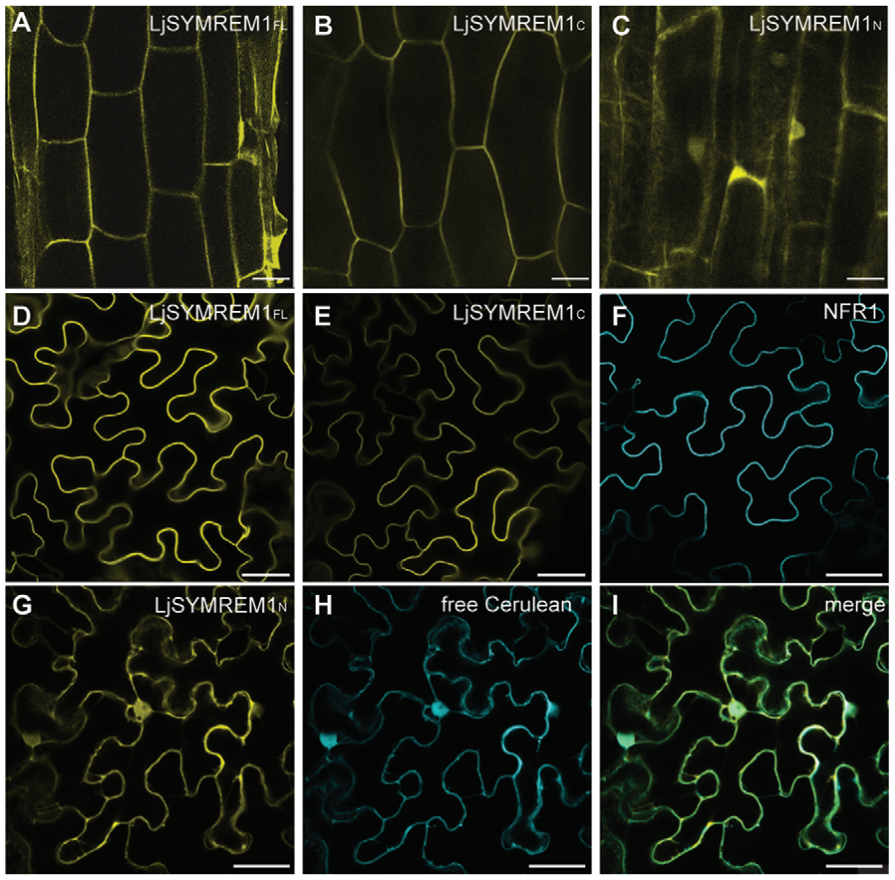
Expression of LjSYMREM1 variants in *L. japonicus* roots and *N. benthamiana* leaves. Clones derived from cDNA of LjSYMREM1 were C-terminally tagged with the mOrange fluorophore and expressed under control of the *Lotus* polyubiquitin promoter in transgenic *L. japonicus* roots (A–C) and as a CaMV-35S promoter-driven construct in leave epidermal cells of *N. benthamiana* (D,E,G). The full-length (FL) protein and the C-terminal region of LjSYMREM1 (LjSYMREM1_C_) are associated to the PM while the N-terminal region (LjSYMREM1_N_) is cytosolic indicated by visible cytoplasmatic strands. In addition NFR1:Cerulean (F) and free Cerulean (H) were expressed in *N. benthamiana* leaves resulting in PM and cytosolic localization, respectively. Bars indicate 200 µm (A–C) and 50 µm (D–I).

The LjSYMREM1 protein is comprised of two main parts, the conserved C-terminal region with a strong prediction for a coiled-coil domain (COILS probability >90%) and the variable N-terminal region. While the C-terminal region (amino acids 79–207; LjSYMREM1_C_) has a predicted globular structure (GlobDoms by Russell/Linding definition), almost only random coils and unfolded structures are predicted for the N-terminal part (amino acids 1–78; LjSYMREM1_N_). Next we identified the domain responsible for PM localization. LjSYMREM1_N_ and LjSYMREM1_C_ regions were individually fused to the mOrange fluorophore and expressed under control of the *Lotus* polyubiquitin promoter in *L. japonicus* hairy roots (Figure 5A–5C). As expected the full-length LjSYMREM1 protein localized to the periphery of root epidermal cells (Figure 5A) indicating membrane association of the protein. This localization was also detected when expressing LjSYMREM1_C_ (Figure 5B) while LjSYMREM1_N_ localized to the cytosol and the nucleus (Figure 5C) indicating that the PM binding motif is located in the C-terminal region of the protein. However, nuclear localization of the LjSYMREM1_N_:mOrange construct was not expected, but due to the small size and the unordered structure of the N-terminal region, the fusion may not interfere with the nuclear import of free fluorophores.

### LjSYMREM1 oligomerizes and interacts with symbiotic RLKs

To understand the roles of the domains we tested interactions between individual LjSYMREM1 domains and other proteins. Since the *in planta* approaches currently require expression of the proteins in a heterologous system such as *N. benthamiana* leaves we first tested whether localizations of these constructs follows those observed in *Lotus* roots. Indeed the full-length protein as well as LjSYMREM1_C_ localized to the PM (Figure 5D–E) as it was also shown for NFR1 (Figure 5F). In contrast, LjSYMREM1_N_ was detected in the cytosol (Figure 5G). Co-localization of LjSYMREM1_N_ with free Cerulean fluorophore protein in *N. benthamiana* leaves confirmed cytosolic localization (Figure 5H–5I).

**Figure 6:**
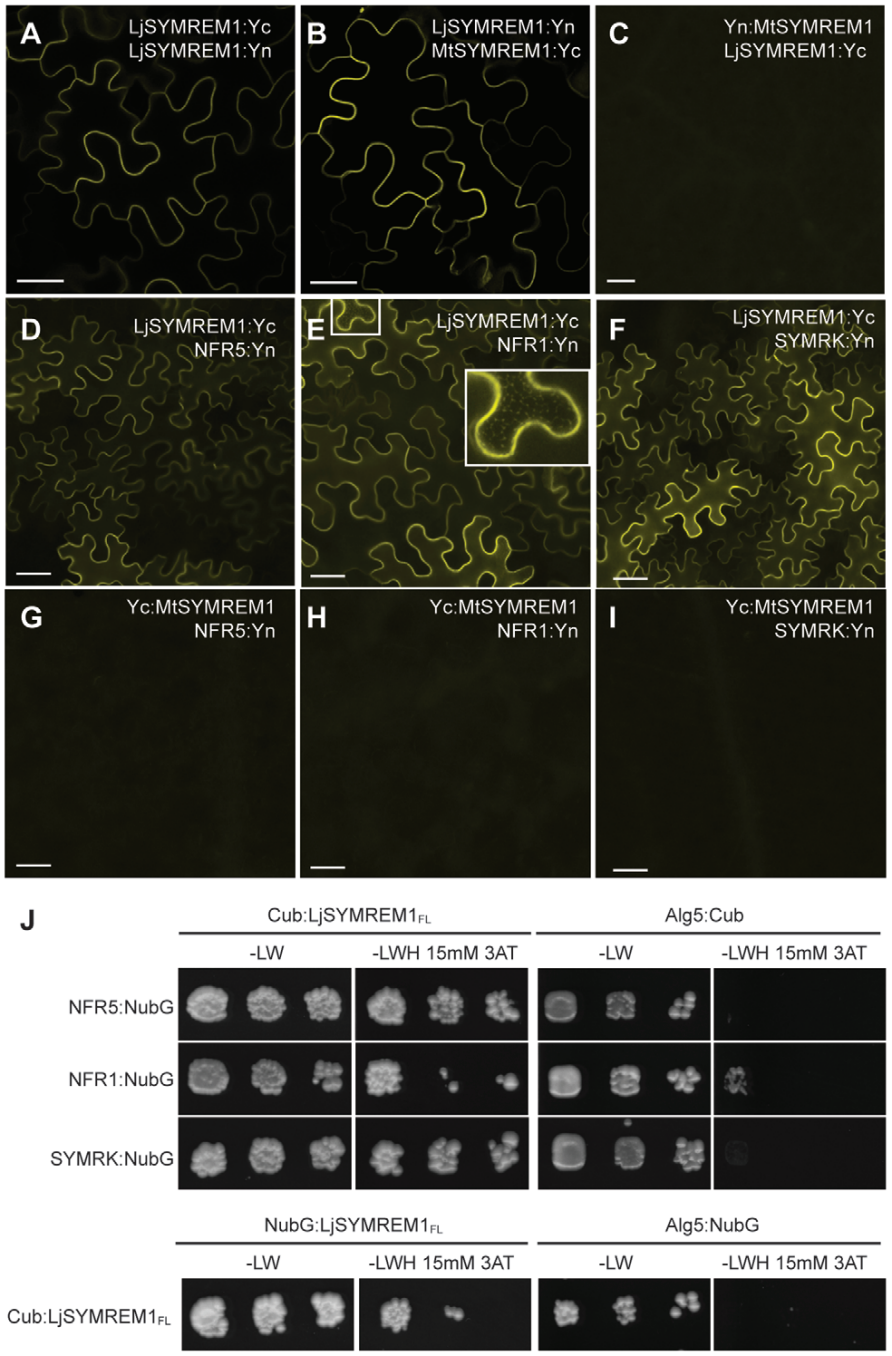
Interactions between LjSYMREM1 and symbiotic RLKs. Bimolecular complementation (BiFC) experiments show that LjSYMREM1 is able to interact with itself and MtSYMREM1 is indicated by the presence of YFP fluorescence (A,B). However, no signal was observed when the MtSYMREM1 protein was N-terminally fused to one half of the YFP protein (C). This demonstrates that overexpression alone is not sufficient to re-assemble the YFP protein. LjSYMREM1 is also able to interact with the three RLKs NFR5, NFR1 and SYMRK (D–F). Bars indicate 40 µm. Occasionally fluorescent foci were observed (E, inset). The yeast split-ubiquitin assay was used to test interactions between full-length LjSYMREM1 itself and the RLKs NFR1, NFR5 and SYMRK (J). The coding regions were fused to the C-terminal half (Cub) and the N-terminal half (NubG) of ubiquitin and interaction was tested on an individual basis. Yeast growth on medium lacking leucine and tryptophan (−LW) shows the presence of both constructs. Interaction was tested on medium additionally lacking histidine (−LWH) that was supplemented with 15 mM 3-amino-1,2,4-triazole (3-AT) to suppress residual levels of endogenous histidine biosynthesis. The yeast resident ER protein Alg5 was used as negative control (Alg5:NubG and Alg5:Cub) (J).

As a proof of concept we then tested for possible interactions between the LjSYMREM1 protein and the symbiotic RLKs NFR5, NFR1 and SYMRK from *L. japonicus* using Bimolecular Fluorescence Complementation (BiFC) (Figure 6A–6I) and the yeast split-ubiquitin system (SUS) (Figure 6J) to assess if LjSYMREM1 exhibits the same interaction patterns as its homolog MtSYMREM1.

For BiFC (also termed split-YFP), we individually fused the proteins to the N- (Y_N_) and C-terminal (Y_C_) halves of YFP and expressed different combinations in leaves of *N. benthamiana* for two days. Interaction between proteins should result in re-assembly of the functional YFP protein and thus in fluorescence at the sites of interaction. Co-expression of LjSYMREM1:Y_C_ and LjSYMREM1:Y_N_ resulted in strong fluorescence in epidermal cells indicating interaction of the proteins (Figure 6A). This is in agreement with the previously reported homo-oligomerization when expressing Y_C_:MtSYMREM1 and Y_N_:MtSYMREM1 together in *N. benthamiana* leaves [15]. When both proteins were C-terminally fused to the individual halves of YFP hetero-oligomerization was also observed between LjSYMREM1 and MtSYMREM1 (Figure 6B). In contrast co-expression of LjSYMREM1:Y_C_ and Y_N_:MtSYMREM1 did not show fluorescence (Figure 6C) presumably since both halves of the YFP protein were physically separated by changing the fusion direction. Thus they served as negative controls. Due to cleavage of the fluorescent tag of a YFP:LjSYMREM1 construct *in planta* (data not shown), reciprocal experiments could not be performed. Next, we fused the *Lotus* RLKs NFR5, NFR1 and SYMRK to the N-terminal half of the YFP protein and co-expressed them together with LjSYMREM1:Y_C_. Interaction between LjSYMREM1 and the RLK proteins was detected in all three cases (Figure 6D–6F). Fluorescence localized to the periphery of the cells indicating PM resident interactions of the proteins. However, expression frequently led to formation of PM associated foci (inlet Figure 6E). Interestingly, no fluorescent signal was detected when these RLKs were co-expressed with the Y_C_:MtSYMREM1 construct (Figure 6G–6I).

To verify the RLK interaction data we used the yeast split-ubiquitin system. Similar to the principle of BiFC the ubiquitin protein was split in two halves. Upon protein interaction re-assembly of the full ubiquitin molecule occurs. The assembled ubiquitin serves as a recognition site for proteolytic cleavage that results in the release of the LexA transcriptional activator that is fused to a VP16 DNA binding domain that are coupled to the C-terminal half (Cub). Diffusion of this construct into the nucleus leads to activation of a HIS3-reporter enabling the yeast to complement its histidine auxotrophy and thus growth on medium lacking histidine. For these assays we generated Cub:LjSYMREM1 fusions while the C-termini of the RLKs were fused to the mutated N-terminal part of ubiquitin (NubG) that is unable to auto-interact with Cub. As negative control we co-expressed the yeast resident ER protein Alg5 as a Cub construct together with the RLKs while Alg5:NubG was used as control to test auto-activation of the reporter system by Cub:LjSYMREM1. Yeast was grown on medium depleted in leucine and tryptophan (−LW) to select for the presence of both plasmids. To select for positive protein interactions these colonies were stamped onto −LWH medium that was additionally depleted in histidine and supplemented by 15 mM 3-amino-1,2,4-triazole (3-AT) to suppress residual levels of endogenous histidine biosynthesis. Yeast growth was sustained when Cub:LjSYMREM1 was co-expressed with the *Lotus* RLKs indicating an interaction between these proteins while no growth was observed when these proteins were co-expressed with the negative controls Alg5:NubG and Alg5:Cub (Figure 6J).

### The C-terminal LjSYMREM1 domain is required for oligomerization and receptor interactions

**Figure 7:**
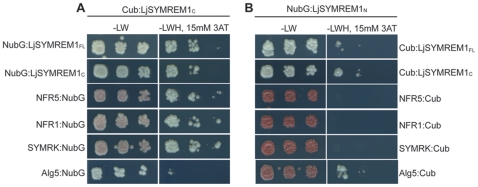
The C-terminal domain of the LjSYMREM1 protein mainly contributed to protein interactions. The yeast split-ubiquitin assay was used to test interactions between the LjSYMREM1 variants and the RLKs NFR1, NFR5 and SYMRK. The coding regions were fused to the C-terminal half (Cub) and the N-terminal half (NubG) of ubiquitin and interaction was tested on an individual basis. Yeast growth on medium lacking leucine and tryptophan (−LW) indicates presence of both constructs. Interaction was tested on medium additionally lacking histidine (−LWH) that was supplemented with 15 mM 3-amino-1,2,4-triazole (3-AT) to suppress residual levels of endogenous histidine biosynthesis. The yeast resident ER protein Alg5 was used as negative control (Alg5:NubG and Alg5:Cub). Yeast growth was sustained on –LWH medium indicating strong interaction of the RLKs and Remorins variants with LjSYMREM1_C_ (A). Weak interaction of LjSYMREM1_N_ with the RLKs and Remorins variants indicates minor or transient contribution of the N-terminal region to protein interactions (B). Pigmentation of yeast indicates severe adenine deficiency as a consequence of lacking interaction. A series of three dilutions (non-diluted, 10^−1^ and 10^−2^) are shown in each panel from left to right).

To assess the individual contributions of both protein regions for Remorin oligomerization LjSYMREM1 (full-length), LjSYMREM1_C_ and LjSYMREM1_N_ were tested on a one-to-one basis. Cytoplasmic localization of LjSYMREM1_N_ (Figure 5C, 5G) only allowed the use of the NubG fusion because the split-ubiquitin assay requires the bait construct (Cub) to be anchored to the plasma membrane, in order to avoid auto-activation of the reporter gene. Co-expression of the LjSYMREM1_C_ construct with full-length LjSYMREM1 resulted in yeast growth under selective conditions indicating that oligomerization of the LjSYMREM1 protein occurs along the C-terminal region of the protein (Figure 7A). Co-transformation of LjSYMREM1_N_ with either LjSYMREM1_C_ or full-length LjSYMREM1 resulted in slight yeast growth on selective conditions to the same extent as observed in the negative controls (Figure 7B). Thus the N-terminal region has no major contribution on LjSYMREM1 oligomerization.

To test domain-specific interactions with the RLKs we co-expressed the different LjSYMREM1 constructs together with the *Lotus* RLKs NFR1, NFR5 and SYMRK. Co-transformation of the LjSYMREM1_C_ construct with the individual RLKs resulted in yeast growth under triple selective conditions indicating a strong interaction (Figure 7A). Since co-expression of the negative control Alg5:NubG resulted in almost no yeast growth it can be concluded that the observed interactions specifically result from the RLK-LjSYMREM1 interaction. In contrast, no interaction was found when these RLKs were co-transformed with LjSYMREM1_N_ (Figure 7B). However, yeast grew on –LWH plates after co-transformation of the RLKs with the positive control Alg5:NubI proving expression of the fusion proteins (Figure S3).

Since both yeast split-ubiquitin and BiFC assays are mostly suitable to qualitatively detect stable protein-interactions we performed fluorescence lifetime imaging microscopy (FLIM) to characterize and quantify interaction by Foerster resonance energy transfer (FRET). We used a Cerulean-mOrange FRET pair, where one protein is fused to the donor fluorophore (Cerulean) while the second protein is fused to mOrange which functions as energy acceptor [24]. FRET occurs when both fluorophores are brought into physical proximity (<10 nm) by interaction of the target proteins. In brief, when measuring FRET by FLIM (FLIM-FRET), the average time electrons of the donor molecule (after photon absorption) stay in the excited state is determined by measuring the exponential ‘decay’ rate by time-resolved measurement of the emitted photons. This is then transformed into ‘fluorescence lifetime’ values. Upon occurrence of FRET, the Cerulean fluorescence lifetime becomes shorter (the ‘decay’ is faster) because the excited donor-electrons drop to the ground state faster, due to the direct energy transfer to the acceptor (mOrange).

For this approach we used the mOrange fusions with LjSYMREM1, LjSYMREM1_C_ and LjSYMREM1_N_ while NFR1, which was taken as a representative RLK, was fused to the Cerulean protein. *N. benthamiana* leaves were co-infiltrated three times independently with *A. tumefaciens* carrying the respective fusion constructs. FLIM-FRET data recording was performed two days post infiltration. For every tested combination (see Table S2), 6–13 lifetime images were collected per tobacco leaf (n = 3–5). To determine the Cerulean lifetime under non-interacting conditions we expressed NFR1:Cerulean alone. To assess a possible effect of acceptor fluorophore over-accumulation on the donor lifetime, NFR1:Cerulean was expressed together with free mOrange. No significant differences between lifetimes of solely expressed NFR1:Cerulean (2.18±0.013 ns) and NFR1:Cerulean/free mOrange (2.16±0.014 ns) were detected (Table S2) indicating that acceptor-accumulation did not have any impact on the donor lifetime. When co-expressing NFR1:Cerulean and LjSYMREM1:mOrange the Cerulean lifetimes were significantly reduced to 1.99±0.022 ns, corresponding to a FRET efficiency of 8.8%. Similar values were obtained when NFR1:Cerulean and LjSYMREM1_C_:mOrange were co-expressed. The observed lifetime decreased to 1.97±0.021 ns with a FRET efficiency of 9.6% clearly indicating physical interaction of NFR1 and the C-terminal region of the LjSYMREM1 protein (Table S2). Moderately but also significantly reduced lifetimes were measured when co-expressing NFR1:Cerulean and LjSYMREM1_N_:mOrange (2.09±0.019 ns; FRET efficiency of 4.1%) (Table S2). These data indicate that primarily the C-terminal region of LjSYMREM1, containing the coiled-coil domain, contributes to NFR1-LjSYMREM1 interaction while the N-terminal region only weakly or transiently interacts with NFR1.

### Phosphorylation of LjSYMREM1 by kinase domains of NFR1 and SYMRK

**Figure 8:**
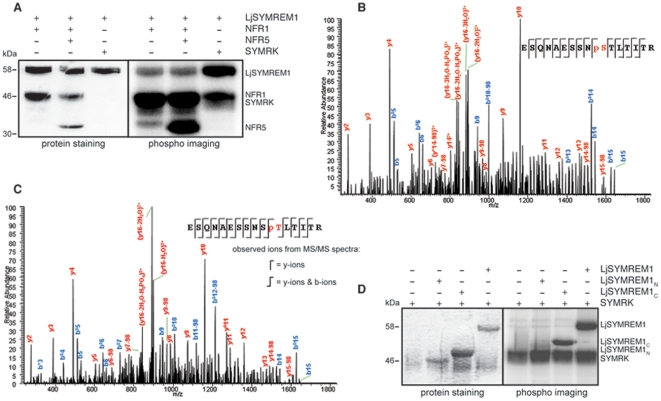
NFR1 and SYMRK kinase domains are able to phosphorylate LjSYMREM1 *in vitro*. Recombinant proteins purified from *E. coli* were tested for phosphorylation *in vitro*. LjSYMREM1 was N-terminally fused to the maltose binding protein (MBP). While NFR1 and NFR5 kinase domains (CD; cytosolic domains of the RLKs were used) were used as untagged proteins, SYMRK-CD contained a His-tag at its C-terminal end. Phosphorylation was visualized by detection of integrated radioactively labeled γ-^32^P-ATP. Both CDs were able to phosphorylate LjSYMREM1 even though NFR1 to a lower extent than SYMRK (A). Autophosphorylation of NFR1 and SYMRK kinase domains as well as trans-phosphorylation of NFR5-CD (inactive) by NFR1 were observed. Presence of NFR5 did not change the level of LjSYMREM1 phosphorylation. Protein staining of the SDS-PAGE shows presence of used proteins. Due to high kinase activity of SYMRK-CD protein amounts used for the assay were decreased to 0.25 µg and thus not visible on the gel (A). Representative MS/MS spectra of phosphorylated peptide ESQNAESSNSpTLTITR (NFR1-LjSYMREM1) (B) and ESQNAESSNpSTLTITR (SYMRK-LjSYMREM1) (C) were obtained when mapping the phosphorylation sites S48 and T49 on the LjSYMREM1 protein, respectively. While SYMRK was able to phosphorylate the C-terminal part of the protein, the LjSYMREM1 N-terminal region alone could not be phosphorylated *in vitro* (D).

As shown above the C-terminal region of the LjSYMREM1 forms a stable interaction with the RLKs while the N-terminal domain may undergo weak or transient interaction. Since Remorins were reported to be phosphorylated *in vivo* [25], [26], [27], [28] we decided to test if the putative transient interactions between the RLKs and the LjSYMREM1_N_ domain is a result of rapidly occurring protein phosphorylation. Contact between proteins should occur along the intracellular region (juxtamembrane region, kinase domain and C-terminal region) of the RLKs since Remorins are anchored to the cytosolic face of the PM [18]. Therefore, we tested if the cytoplasmic domains (CDs) of these symbiotic RLKs are able to phosphorylate LjSYMREM1 *in vitro*. It should be noticed that NFR5 is a pseudokinase that lacks several kinase subdomains including the activation loop and has recently been shown to lack kinase activity *in vitro* [1], [29]. Purified LjSYMREM1 was tested with the recombinant CDs of NFR1, NFR5 and SYMRK. As illustrated in Figure 8A SYMRK was able to phosphorylate LjSYMREM1. A clear, but weaker, phosphorylation of LjSYMREM1 was found when the protein was incubated with NFR1 alone or in the presence of both NFR1 and NFR5. No phosphorylation was observed when purified MBP protein was used as substrate of NFR1, demonstrating that the phosphorylation of LjSYMREM1 did not derive from phosphorylation of the MBP tag (Figure S4).

To map the phosphorylation sites on the LjSYMREM1 protein, phosphorylation reactions were repeated under non-radioactive conditions, LjSYMREM1 bands were excised from the SDS–polyacrylamide gel and tandem mass spectrometric analysis (MS/MS) was performed. Phosphorylated residues were neither detected on the LjSYMREM1 nor on the MBP proteins in the absence of NFR1 and SYMRK, indicating the absence of LjSYMREM1 and MBP phosphorylation by bacterial kinases. MS/MS analysis of LjSYMREM1 phosphorylated by NFR1 and SYMRK revealed that serine S48 and threonine T49 located within the N-terminal region of the SYMREM1 protein, were phosphorylated by these kinase domains, respectively (Figure 8B–8C). The obtained Mascot score were 54 for the T49 and 57 for phosphorylation of the S48 while the MS/MS spectra did not permit us to rule out that only one of the residues was phosphorylated. However, bioinformatic predictions (NetPhos2.0) indicate high P-site probabilities for S48 (0.994) while T49 is unlikely to represent an active P-site (score 0.180). These results are also supported by the fact that S48 is conserved in both MtSYMREM1 and LjSYMREM1 while T49 can only be found in the *Lotus* protein. Despite a high LjSYMREM1 sequence coverage (92%) the possibility cannot be excluded that S91, S130 and/or T131 may also be phosphorylated, as the 89-VESQK-93 and 127-KASTQAK-134 peptide fragments could not be detected during the experiments.

To test this we purified recombinantly expressed LjSYMREM1_C_ and LjSYMREM1_N_ proteins and used them independently in a kinase assay with SYMRK that was shown to be the strongest phosphorylating kinase (Figure 8A). Indeed SYMRK could phosphorylate LjSYMREM1_C_ indicating the presence of an additional phosphorylation site in the C-terminal region. Interestingly, when LjSYMREM1_N_ was co-incubated with SYMRK, no phosphorylation of this domain was detected (Figure 8D) suggesting that the C-terminal region form a stable kinase-LjSYMREM1 interaction that subsequently allows phosphorylation of the Remorin N-terminal domain.

## Discussion

Despite the fact that most signaling proteins involved in RNS are highly conserved between *M. truncatula* and *L. japonicus*, SYMREM1 proteins from legumes show a remarkable variability in their N-terminal regions (Figure 1; Table S1) indicating either high evolutionary pressure on group 2 N-terminal regions or dispensability of the domain. Given the emerging roles of Remorins to act as novel modulators in plant signaling cascades we therefore characterized the LjSYMREM1 protein from *Lotus japonicus* in more detail with the aim to determine its spatio-temporal regulation and domains within the protein that contribute to the RLK-Remorin complex formation. The finding that purified NFs were sufficient to induce the promoter in root epidermal and cortical cells and that *LjSYMREM1* expression followed nodule primordium formation supports putative role of the protein during initial stages of rhizobial infection. Epidermal activation of the promoter was entirely abolished during nodule organogenesis and infection while GUS staining was continuously observed in infected cells of mature determinate nodules of *Lotus* (Figure 3) where the native LjSYMREM1 protein strongly accumulates (Figure 4). Whether the spatial expression of the *Lotus* RLKs NFR1, NFR5 and SYMRK matches the profile of *LjSYMREM1* during later stages of the nodulation process remains to be studied. However, continuous expression of the orthologous RLKs from *M. truncatula* in nodule primordia has been shown while in nodules transcripts have only been detected in the infection zone [6], [9], [30]. These data suggest roles of these RLKs also during later stages of infection. Whether the receptors are also present on symbiosome membranes has not been reported, yet. LjSYMREM1 is also present on trans-cellular infection threads that connect infected cells in mature nodules (Figure S2B). These data complement the findings that MtSYMREM1 localizes to nodular infection threads within the infection zone (zone II) [15], however no MtSYMREM1 protein was detected on remnant trans-cellular infection threads in the fixation zone (zone III) of *Medicago* nodules (Ton Timmers, LIPM Toulouse, *personal communication*).

In order to better understand the biology of Remorins and the structural requirements for RLK-Remorin interactions we separated the N- and C-terminal regions according to the presence of the coiled-coil domain in the C-terminal part and the lack of sequence conservation compared to other Remorins in the N-terminal region. As expected, due to the fact that PM association has been suggested for the entire Remorin family, the solely expressed C-terminal region localized to the plasma membrane (Figure 5) while the N-terminal region does not contribute to the subcellular localization of LjSYMREM1. Furthermore data presented here show that both, Remorin oligomerization and interaction with RLKs are mainly mediated also by the C-terminal part as shown in yeast (Figure 6), by FLIM analysis (Table S2) and by *in vitro* kinase assays (Figure 8B). The lack of fluorescence in the BiFC assay when co-expressing Yc:MtSYMREM1 and LjSYMREM1:Yn (Figure 6) indicates that Remorins may assemble in a parallel fashion leading to a physical distance of the split YFP halves and thus the lack of fluorescent signal. Whether phosphorylation of C-terminal residues is required for oligomerization remains to be studied. However, the fact that Yc:MtSYMREM1 does not interact with any of the *Lotus* RLKs (Figure 6) may also suggest that the N-terminal region of the both homologs has a steric impact on these interactions. Thus the function of the N-terminal region remains to be studied in detail and will likely provide further functional insights into SYMREM1 function.

Coiled-coil motifs are well known domains required for protein-protein interactions and several CCD containing proteins involved in cellular signaling processes have been described [31], [32]. This domain has been previously hypothesized to be involved in Remorin oligomerization [33]. Since PM association of LjSYMREM1 is mediated by residues in the C-terminal region (Figure 5) we assume that LjSYMREM1_C_ tightly associates with the kinase- and/or juxtamembrane domains of the receptors in close proximity to the PM. However, our FLIM data indicate that the N-terminal region weakly or transiently interacts with the RLK cytoplasmic domains (Table S2). In line with this we mapped the NFR1 and SYMRK phosphorylation site (S48/T49) to the N-terminal region of LjSYMREM1 (Figure 8). This phosphorylation possibly requires formation of a stable receptor-Remorin complex *in vivo*. It remains to be investigated whether phosphorylation of S48 induces a conformational change in the N-terminal region of the protein that allows interaction with other proteins and how specificity for recognition of interaction partners is achieved.

Molecular scaffold proteins are able to recruit proteins in membrane subdomains such as membrane rafts and facilitate assembly of multi-component signaling complexes. We hypothesize that LjSYMREM1 also serves such function. However, the fact that NFR1 and NFR5 are able to interact with each other in the absence of LjSYMREM1 at least when heterologously overexpressed in *N. benthamiana* [29] implies that the protein might be required for recruitment of RLKs into membrane rafts and to facilitate complex assembly in these subdomains. The fact that a large-scale proteomic study of *M. truncatula* membrane raft localized proteins did not identify LYK3, NFP or DMI2 in membrane rafts [34] may rather reflect low abundance of the RLK proteins. However it was recently nicely shown that LYK3 localizes to mobile membrane micro-domains in *Medicago* root hairs. Application of Nod Factors immobilized these foci and led to co-localization with the flotillin protein FLOT4 [35] that has been previously shown to be required during rhizobial infections [36]. It remains an intriguing question for the future if direct interactions between symbiotic RLKs and flotillins together with remorin proteins occur.

## Materials and Methods

### Phylogenetic and sequence analyses

Alignments and phylogenetic trees were computed using the CIPRES web-portal. Alignments were computed with MAFFT 6.822 (JTT matrix, E-INS-i setting) and RAxML 7.2.7 for fast maximum likelihood analyses [37]. For RAxML, the JTT PAM matrix for amino acid substitutions was chosen and the GTRGAMMA model was used for both, the bootstrapping phase and the final tree inference model, with 1000 bootstraps.

The 147 Remorin protein sequences available from public databases were analyzed to study their relationship, using 101 unambiguously aligned amino acid positions of the conserved C-terminal region. A second dataset contained 96 aligned sequences of group 2 Remorins only, allowing the analysis of 172 positions.

For sequence comparisons (Table S1 A–C) sequences were pairwise aligned using the EMBOSS Stretcher Algorithm (http://www.ebi.ac.uk/Tools/psa/emboss_stretcher/).

### Plant Growth, Hairy Root Transformation and Stable Transformation

Transgenic roots were generated using *Agrobacterium rhizogenes* AR1193 [38] carrying the relevant construct. Roots of all plants were removed and seedlings were dipped into *Agrobacterium* suspension. Transformed plants were plated onto Gamborg's B5 medium [39], incubated in dark for 2 days before being grown at 24°C (8 h dark/16 h light, 60% humidity). Removal of Agrobacteria was achieved by transferring plants on Gamborg's B5 medium containing Cefotaxim 5 days after transformation.

Four weeks after transformation, plants were infected with *Mesorhizobium loti* MAFF 303099 (expressing DsRed fluorophore) and grown in glass jars on sand-vermiculite (1∶1) mixture for the time indicated in the individual experiments.

Stable transformation of *Lotus japonicus* MG20 wild-type plants was performed as described earlier [40] with slight modifications. The offspring of primary transformed plants (T2) was selected by hygromycin resistance and grown in glass jars on sand-vermiculite and were infected with *M. loti* MAFF 303099.

### Promoter Analysis - Histochemical GUS-Staining/ß-Glucuronidase Assay

For analysis of promoter activity a 975 bp fragment upstream of the translational start codon was cloned into pBI101 binary vector and fused to the *uidA* (GUS) reporter gene. Transgenic roots of composite plants carrying the 975 bp construct as well as the empty vector - as negative control - were harvested periodically one week after inoculation with *M. loti* MAFF 303099 and incubated in GUS-staining solution (0.1 M NaPO_4_; 1 mM EDTA; 1 mM K_3_Fe(CN)_6_; 1 mM K_4_Fe(CN)_6_; 1% Triton-×100; 1 mM X-Gluc) at 37°C in dark for 5 hours.

For imaging nodules were embedded in 5% Low-melt Agarose and sectioned via a Leica VT1000s Vibratome into 100 µm thick sections. For GUS-staining (Figure S1) nodules were sectioned prior to GUS staining and staining was performed over-night.

### Cloning and Constructs

All cloning steps (if not specifically indicated) were performed using Gateway technology. Created entry clones were verified by sequencing. All LjSYMREM1 and RLK constructs are based on cDNA templates until stated differently. SYMRK constructs that were used for *in planta* expression derived from genomic based clones.

For BiFC vectors were used as described earlier [15]. For FLIM analysis p35S-GW-Cerulean-nos and p35S-GW-mOrange-nos [41] vectors were used.

To analyze the localizations of LjSYMREM1 in the homologous *L. japonicus* background, we generated C-terminal fusions of the different LjSYMREM1 constructs to mOrange fluorophore in a vector that was described earlier [23] where the mOrange fluorophore was inserted after the recombination cassette.

To generate a stable *L. japonicus* line for protein localization expressed under its native promoter, we fused a 975 bp native promoter sequence and the full-length genomic sequence of LjSYMREM1 C-terminally to the eYFP fluorophore using the pH7YWG2.0 vector (modified after [42]) after removal of the CaMV-35S-promoter.

### Protein Extraction and Western-blot Analysis

Total protein extraction was performed from transgenic *Lotus* roots expressing LjSYMREM1-mOrange under control of the *Lotus* polyubiquitin promoter and from roots of the stable transgenic plants expressing LjSYMREM1-YFP under its endogenous promoter. Roots were ground in liquid nitrogen and homogenized in denaturating buffer (10 mM EDTA, 50 mM Hepes, 150 mM NaCl, 10% Sucrose, 5 M Urea, 2 M Thiourea, 1% Triton-X 100, 1% SDS, 2 mM DTT, plant protease inhibitor cocktail from Sigma) and incubated for 1 hour at 37°C. Proteins were separated on a 12% SDS gel and transferred overnight at 4°C onto PVDF membranes. Membranes were blocked in 5% milk in TBS (with 0.1% Tween 20) and incubated overnight at 4°C with primary antibody. For detection of mOrange and the YFP fluorophore primary α-DsRed (rabbit, polyclonal; 1∶5000) and primary α-GFP (mouse, monoclonal; 1∶5000) antibodies were used, respectively.

### Yeast two-hybrid interaction assay

We used the yeast split-ubiquitin system (SUS) for testing protein-protein interactions using the NMY32 yeast strain. The Remorin constructs were cloned into the bait vector pBT3-N (Cub – C-terminal half of the Ubiquitin molecule – N-terminal fusion to the protein) and into the prey vector pDSL-Nx (NubG – mutated N-terminal Ubiqutin domain - N-terminal fusion to the protein) using SfiI restriction sites. RLK bait constructs were cloned into pTMBV4 (NFR1, SYMRK) and in pBT3-C (NFR5). For using the RLKs as prey constructs genes were cloned into pDL2xN. Co-transformations to investigate and confirm interactions and crude protein extraction were performed as described by the manufacture (DUALsystem). Transformants were tested for interactions on SD (0.67% yeast nitrogen base, 2% glucose, 2% Bacto-agar and amino acid mix) without the appropriate auxotrophic markers and in the presence of 15 mM 3-amino-1,2,4-triazole (3-AT) in different dilution series: ND (non-diluted), 10^−1^, 10^−2^ up to 10^−5^.

### BiFC studies and FLIM-FRET analysis

BiFC experiments were performed as described earlier [29]. Imaging was performed with a spectral TCS SP5 MP confocal laser-scanning microscope (Leica Microsystems, Mannheim, Germany) using an argon laser at an excitation wavelength of 514 nm. The water immersion objective lens HCX PL APO 20.0×0.70 IMM UV was used for imaging tobacco epidermal cells for confocal imaging and FLIM analysis.

For FLIM-FRET analysis *Agrobacterium*-infiltration of tobacco leaves was performed as described above using *A. tumefaciens* GV3101 C58 carrying the respective constructs. For confocal laser scanning microscopy (CLSM) Cerulean fusion proteins were excited with a 405 nm diode laser, whereas mOrange fusion proteins were excited with a 514 nm argon laser line [41]. Cerulean fluorescence emission was detected between 485 and 535 nm, whereas mOrange fluorescence emission was detected between 545 and 600 nm. For spectroscopic analysis, the emission spectra of Cerulean and mOrange were recorded by λ-scanning between 450–590 nm and 540–720 nm, respectively.

For FLIM-FRET measurements, multiphoton (MP) excitation was used. Cerulean was excited with 810-nm light using a Spectra Physics Ti:Sapphire Mai Tai laser running at 80mhz with 1.2 ps pulse lengths. A FLIM PMT detector build in the spectral scanhead of the above mentioned microscope (Becker & Hickl [B&H] FLIM setup, implemented by Leica Microsystems, Mannheim, Germany) was used for time resolved photon detection for 5 min in 64 scanning cycles (≈5 s/cycle) at a spatial resolution of 256×256 pixel, using the B&H photon counting software TCSPC 2.80. The MP excitation laser-power used was at setting that resulted in less then 10% photobleaching over the 5 min measuring time.

Selected magnified areas of the cells were then subjected to analyses performed with the B&H SPCImage software. Significance levels were calculated by student's t-test (with *p*<0.01 being significantly different).

### *In Vitro* Phosphorylation Assay

For this study the different LjSYMREM1 constructs were fused to the C-terminus of *E. coli* maltose-binding protein MalE (MBP) in pKM596 using the pENTR/TEV/D-TOPO entry clones. Proteins were expressed in *E. coli* Rosetta cells upon induction with 0.5 mM IPTG, cells were harvested after incubation at 21°C for 6 hours. Proteins were purified by amylose resin affinity chromatography (binding buffer: 20 mM Tris-HCl pH 7.4, 200 mM NaCl, 10 mM ß-mercaptoethanol, 1 mM EDTA and 1 protease inhibitor tablet per 200 ml) and eluted samples (elution buffer: 20 mM Tris-HCl pH 7.4, 200 mM NaCl, 10 mM ß-mercaptoethanol, 1 mM EDTA, 10 mM maltose and 1 protease inhibitor tablet per 200 ml) were tested on 10% SDS gel.

Purified MBP-LjSYMREM1, MBP-LjSYMREM1c and MBP-LjSYMREM1_N_ were incubated with the respective kinases (NFR1-CD (residues 254–622), NFR5-CD (residues 276–596), SYMRK-CD (residues 541–923)) for 45 min in kinase buffer (10 mM HEPES pH 7.4; 2 mM MgCl_2_; 2 mM MnCl_2_; 0.2 mM DTT; 2 µM ATP). For radioactive labeling proteins were incubated as described above in the presence 10 µCi [**γ**-^32^P]ATP.

### In Gel Digestion

Protein bands were excised from the SDS gels and bands were cut into 11 mm^3^ pieces and destained with 30% acetonitrile. The samples were reduced with 10 mM dithiothreitol in 25 mM ammonium bicarbonate for 30 min at 56°C and subsequently alkylated with 55 mM iodoacetamide in 25 mM ammonium bicarbonate for 45 min at room temperature in the dark. Then, the gel pieces were washed with water and ACN and dried under vacuum. Finally, proteins were digested with trypsin (1∶20, w/w) in 25 mM ammonium bicarbonate (pH 8.0) overnight at 37°C.

### Phosphopeptide enrichment with TiO_2_ micro-column

Peptides were extracted from the gel by 5% FA 30% ACN. Phosphopeptides were enriched using micro-column as described earlier [43], [44]. A small C8 plug (3 M C8 disk) was made using a HPLC syringe and placed at the constricted end of the Geloader tip. The TiO_2_ material in 100% ACN was packed on top of the C8 plug. The dried peptides were resuspended with 50 µl of TiO_2_ loading buffer and directly loaded onto the TiO_2_ micro-column. After washing one time with 20 µl loading buffer and twice with 20 µl washing buffer (80% ACN, 1% TFA), the bound peptides were eluted with 20 µl 1 M NH_3_·H_2_O and 5 µl of 0.5 M NH_3_·H_2_O in 30% ACN. The elution was acidified with 1 µl 100% formic acid and dried prior to LC-MS analysis.

### LC MS/MS Analysis and Data Interpretation

LC-MS/MS analysis was performed using a nanoliter flow EasyLC system (Thermo Fisher Scientific, Odense, Denmark) interfaced to an LTQ-Orbitrap XL mass spectrometer (Thermo Fisher Scientific, Bremen, Germany) as described earlier [29], [45].

## Supporting Information

Figure S1 **Sections of nodules expressing an _pLjSYMREM1_:GUS construct.** The construct was expressed in *L. japonicus* roots and GUS staining was performed 24 hours after Nod Factor application (A) and 21 dpi with *M. loti* (B). GUS staining was found in outer and inner root cortical cells (A), infected cells of nodules containing nitrogen-fixing bacteroids as well as in outer parenchyma cells that are not infected by the bacteria (B). Root material and nodules were sectioned after or prior to GUS staining that was performed over-night, respectively. Scale bars indicate 25 µm (A) and 500 µm (B).
(TIF)

Figure S2 **LjSYMREM1:YFP localizes to the symbiosome membrane and to nodular infection threads.** A genomic construct consisting of the *LjSYMREM1* native promoter and the *LjSYMREM1* gene was fused to YFP. Roots were inoculated with *M. loti* MAFF303099 and three week old nodules of stable transgenic T2 plants were analyzed. Infected cells were disrupted by mechanical force to separate symbiosomes. Individual symbiosomes showed clear YFP fluorescence indicating presence of LjSYMREM1 on the symbiosome membrane (A). YFP fluorescence was also detected on nodular infection thread remnants that are found in between infected cells (B). Bars indicate 5 µm (A) and 10 µm (B).
(TIF)

Figure S3 **All membrane-anchored clones were expressed in the yeast split-ubiquitin system.** The NubI tag is able to reconstitute together with Cub to the full-length ubiquitin and thus activates expression of the *HIS3*-reporter. Yeast growth on medium lacking leucine and tryptophan (−LW) shows the presence of both constructs. Interaction was tested on medium additionally lacking histidine (−LWH) that was supplemented with 15 mM 3-amino-1,2,4-triazole (3-AT) to suppress residual levels of endogenous histidine biosynthesis.
(TIF)

Figure S4 **NFR1 and SYMRK kinase domains are unable to phosphorylate maltose binding protein (MBP).** MBP was recombinantly expressed and purified. Since no phosphorylation of MBP was detected in kinase assays it can be concluded that phosphorylation that was observed with MBP-LjSYMREM1 does not derive from MBP phosphorylation.
(TIF)

Table S1 **Group 2 remorins exhibit unusual sequence diversity in their N-terminal region.** Sequence comparison of full-length (overall), N- and C-terminal protein sequences of legume remorins that were found to be most closely related to each other (Figure 1) revealed that sequence conservation of the C-terminal region is in accordance with similarities of other legumes signaling proteins (Table S1B) while the N-terminal region is unusually diverse (Table S1A).
(DOCX)

Table S2 **Testing interaction between LjSYMREM1 domains and NFR1 by FLIM analysis.** LjSYMREM1:mOrange and NFR1:Cerulean were co-expressed in *N. benthamiana* leaves under control of the CaMV 35S-promoter. Shorter Cerulean lifetimes indicate interaction between the proteins. Strong interaction was observed between NFR1 and LjSYMREM1_FL_/LjSYMREM1_C_ while mild but significant reduction in lifetime was also observed between NFR1 and LjSYMREM1_N_. Numeric values are provided in the table inset. Significance levels were calculated by student's t-test (with *p*<0.01 being significantly different). Free mOrange was co-expressed with NFR1:Cerulean to demonstrate that simple protein accumulation by over-expression of the acceptor fluorophore is not sufficient to reduce donor lifetimes.
(PDF)

## References

[1] Madsen EB, Madsen LH, Radutoiu S, Olbryt M, Rakwalska M (2003). A receptor kinase gene of the LysM type is involved in legume perception of rhizobial signals.. Nature.

[2] Radutoiu S, Madsen LH, Madsen EB, Felle HH, Umehara Y (2003). Plant recognition of symbiotic bacteria requires two LysM receptor-like kinases.. Nature.

[3] Radutoiu S, Madsen LH, Madsen EB, Jurkiewicz A, Fukai E (2007). LysM domains mediate lipochitin-oligosaccharide recognition and *Nfr* genes extend the symbiotic host range.. EMBO J.

[4] Oldroyd GE, Downie JA (2008). Coordinating nodule morphogenesis with rhizobial infection in legumes.. Annu Rev Plant Biol.

[5] Popp C, Ott T (2011). Regulation of signal transduction and bacterial infection during root nodule symbiosis.. Curr Opin Plant Biol.

[6] Arrighi JF, Barre A, Ben Amor B, Bersoult A, Soriano LC (2006). The *Medicago truncatula* lysin motif-receptor-like kinase gene family includes NFP and new nodule-expressed genes.. Plant Physiol.

[7] Smit P, Limpens E, Geurts R, Fedorova E, Dolgikh E (2007). *Medicago* LYK3, an entry receptor in rhizobial nodulation factor signaling.. Plant Physiol.

[8] Limpens E, Franken C, Smit P, Willemse J, Bisseling T (2003). LysM domain receptor kinases regulating rhizobial Nod factor-induced infection.. Science.

[9] Bersoult A, Camut S, Perhald A, Kereszt A, Kiss GB (2005). Expression of the *Medicago truncatula DM12* gene suggests roles of the symbiotic nodulation receptor kinase in nodules and during early nodule development.. Mol Plant Microbe Interact.

[10] Limpens E, Mirabella R, Fedorova E, Franken C, Franssen H (2005). Formation of organelle-like N2-fixing symbiosomes in legume root nodules is controlled by DMI2.. Proc Natl Acad Sci U S A.

[11] Stracke S, Kistner C, Yoshida S, Mulder L, Sato S (2002). A plant receptor-like kinase required for both bacterial and fungal symbiosis.. Nature.

[12] Madsen LH, Tirichine L, Jurkiewicz A, Sullivan JT, Heckmann AB (2010). The molecular network governing nodule organogenesis and infection in the model legume *Lotus japonicus*.. Nat Commun.

[13] Gage DJ (2002). Analysis of infection thread development using Gfp- and DsRed-expressing *Sinorhizobium meliloti*.. J Bacteriol.

[14] Gage DJ (2004). Infection and invasion of roots by symbiotic, nitrogen-fixing rhizobia during nodulation of temperate legumes.. Microbiology and Molecular Biology Reviews.

[15] Lefebvre B, Timmers T, Mbengue M, Moreau S, Herve C (2010). A remorin protein interacts with symbiotic receptors and regulates bacterial infection.. Proc Natl Acad Sci U S A.

[16] Raffaele S, Mongrand S, Gamas P, Niebel A, Ott T (2007). Genome-wide annotation of remorins, a plant-specific protein family: evolutionary and functional perspectives.. Plant Physiol.

[17] Kistner C, Winzer T, Pitzschke A, Mulder L, Sato S (2005). Seven *Lotus japonicus* genes required for transcriptional reprogramming of the root during fungal and bacterial symbiosis.. Plant Cell.

[18] Raffaele S, Bayer E, Lafarge D, Cluzet S, German Retana S (2009). Remorin, a *Solanaceae* protein resident in membrane rafts and plasmodesmata, impairs potato virus X movement.. Plant Cell.

[19] Jarsch IK, Ott T (2011). Perspectives on remorin proteins, membrane rafts, and their role during plant-microbe interactions.. Mol Plant Microbe Interact.

[20] Colebatch G, Desbrosses G, Ott T, Krusell L, Montanari O (2004). Global changes in transcription orchestrate metabolic differentiation during symbiotic nitrogen fixation in *Lotus japonicus*.. Plant Journal.

[21] Hogslund N, Radutoiu S, Krusell L, Voroshilova V, Hannah MA (2009). Dissection of symbiosis and organ development by integrated transcriptome analysis of *Lotus japonicus* mutant and wild-type plants.. PLoS One.

[22] Heidstra R, Geurts R, Franssen H, Spaink HP, Van Kammen A (1994). Root Hair Deformation Activity of Nodulation Factors and Their Fate on *Vicia sativa*.. Plant Physiol.

[23] Maekawa T, Kusakabe M, Shimoda Y, Sato S, Tabata S (2008). Polyubiquitin promoter-based binary vectors for overexpression and gene silencing in *Lotus japonicus*.. Mol Plant Microbe Interact.

[24] Koushik SV, Vogel SS (2008). Energy migration alters the fluorescence lifetime of Cerulean: implications for fluorescence lifetime imaging Forster resonance energy transfer measurements.. J Biomed Opt.

[25] Benschop JJ, Mohammed S, O'Flaherty M, Heck AJ, Slijper M (2007). Quantitative phosphoproteomics of early elicitor signaling in Arabidopsis.. Mol Cell Proteomics.

[26] Farmer EE, Moloshok TD, Saxton MJ, Ryan CA (1991). Oligosaccharide signaling in plants. Specificity of oligouronide-enhanced plasma membrane protein phosphorylation.. J Biol Chem.

[27] Reymond P, Kunz B, Paul-Pletzer K, Grimm R, Eckerskorn C (1996). Cloning of a cDNA encoding a plasma membrane-associated, uronide binding phosphoprotein with physical properties similar to viral movement proteins.. Plant Cell.

[28] Widjaja I, Naumann K, Roth U, Wolf N, Mackey D (2009). Combining subproteome enrichment and Rubisco depletion enables identification of low abundance proteins differentially regulated during plant defense.. Proteomics.

[29] Madsen EB, Antolin-Llovera M, Grossmann C, Ye J, Vieweg S (2011). Autophosphorylation is essential for the in vivo function of the *Lotus japonicus* Nod factor receptor 1 and receptor-mediated signalling in cooperation with Nod factor receptor 5.. Plant J.

[30] Mbengue M, Camut S, de Carvalho-Niebel F, Deslandes L, Froidure S (2010). The *Medicago truncatula* E3 ubiquitin ligase PUB1 interacts with the LYK3 symbiotic receptor and negatively regulates infection and nodulation.. Plant Cell.

[31] Rairdan GJ, Collier SM, Sacco MA, Baldwin TT, Boettrich T (2008). The coiled-coil and nucleotide binding domains of the Potato Rx disease resistance protein function in pathogen recognition and signaling.. Plant Cell.

[32] Skirpan AL, McCubbin AG, Ishimizu T, Wang X, Hu Y (2001). Isolation and characterization of kinase interacting protein 1, a pollen protein that interacts with the kinase domain of PRK1, a receptor-like kinase of petunia.. Plant Physiol.

[33] Bariola PA, Retelska D, Stasiak A, Kammerer RA, Fleming A (2004). Remorins form a novel family of coiled coil-forming oligomeric and filamentous proteins associated with apical, vascular and embryonic tissues in plants.. Plant Mol Biol.

[34] Lefebvre B, Furt F, Hartmann MA, Michaelson LV, Carde JP (2007). Characterization of lipid rafts from *Medicago truncatula* root plasma membranes: a proteomic study reveals the presence of a raft-associated redox system.. Plant Physiol.

[35] Haney CH, Riely BK, Tricoli DM, Cook DR, Ehrhardt DW (2011). Symbiotic Rhizobia Bacteria Trigger a Change in Localization and Dynamics of the *Medicago truncatula* Receptor Kinase LYK3.. Plant Cell.

[36] Haney CH, Long SR (2010). Plant flotillins are required for infection by nitrogen-fixing bacteria.. Proc Natl Acad Sci U S A.

[37] Stamatakis A, Hoover P, Rougemont J (2008). A rapid bootstrap algorithm for the RAxML Web servers.. Syst Biol.

[38] Stougaard J, Petersen TE, Marcker KA (1987). Expression of a Complete Soybean Leghemoglobin Gene in Root-Nodules of Transgenic *Lotus-Corniculatus*.. Proceedings of the National Academy of Sciences of the United States of America.

[39] Gamborg OL, Miller RA, Ojima K (1968). Nutrient requirements of suspension cultures of soybean root cells.. Exp Cell Res.

[40] Lombari P, Ercolano E, El Alaoui H, Chiurazzi M, Márquez AJ, Stougaard J, Udvardi M, Parniske M, Spaink H (2005). *Agrobacterium-*mediated in vitro transformation.. *Lotus japonicus* handbook.

[41] Bayle V, Nussaume L, Bhat RA (2008). Combination of novel green fluorescent protein mutant TSapphire and DsRed variant mOrange to set up a versatile in planta FRET-FLIM assay.. Plant Physiol.

[42] Karimi M, De Meyer B, Hilson P (2005). Modular cloning in plant cells.. Trends Plant Sci.

[43] Larsen MR, Thingholm TE, Jensen ON, Roepstorff P, Jorgensen TJ (2005). Highly selective enrichment of phosphorylated peptides from peptide mixtures using titanium dioxide microcolumns.. Mol Cell Proteomics.

[44] Thingholm TE, Jorgensen TJ, Jensen ON, Larsen MR (2006). Highly selective enrichment of phosphorylated peptides using titanium dioxide.. Nat Protoc.

[45] Ye J, Zhang X, Young C, Zhao X, Hao Q (2010). Optimized IMAC-IMAC protocol for phosphopeptide recovery from complex biological samples.. J Proteome Res.

