# Supplementary figures and images for: Functional Domain Analysis of the Remorin Protein LjSYMREM1 in Lotus japonicus
Source: PLoS One. 2012 Jan 23;7(1):e30817. doi: 10.1371/journal.pone.0030817 (PMC3264624; doi:10.1371/journal.pone.0030817)

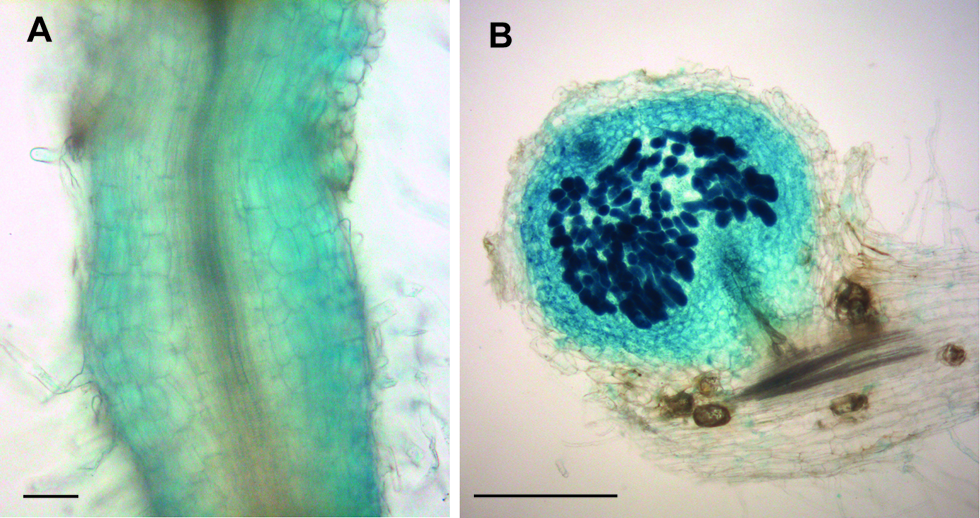

Supplement: Figure S1 — Sections of nodules expressing an pLjSYMREM1:GUS construct. The construct was expressed in L. japonicus roots and GUS staining was performed 24 hours after Nod Factor application (A) and 21 dpi with M. loti (B). GUS staining was found in outer and inner root cortical cells (A), infected cells of nodules containing nitrogen-fixing bacteroids as well as in outer parenchyma cells that are not infected by the bacteria (B). Root material and nodules were sectioned after or prior to GUS staining that was performed over-night, respectively. Scale bars indicate 25 µm (A) and 500 µm (B). (TIF) [file pone.0030817.s001.tif]

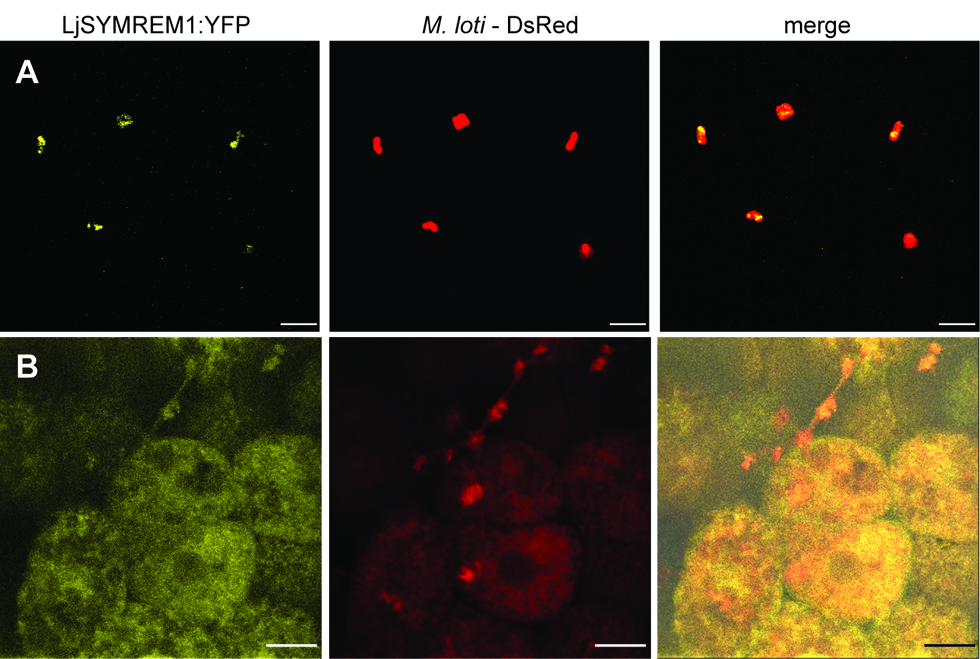

Supplement: Figure S2 — LjSYMREM1:YFP localizes to the symbiosome membrane and to nodular infection threads. A genomic construct consisting of the LjSYMREM1 native promoter and the LjSYMREM1 gene was fused to YFP. Roots were inoculated with M. loti MAFF303099 and three week old nodules of stable transgenic T2 plants were analyzed. Infected cells were disrupted by mechanical force to separate symbiosomes. Individual symbiosomes showed clear YFP fluorescence indicating presence of LjSYMREM1 on the symbiosome membrane (A). YFP fluorescence was also detected on nodular infection thread remnants that are found in between infected cells (B). Bars indicate 5 µm (A) and 10 µm (B). (TIF) [file pone.0030817.s002.tif]

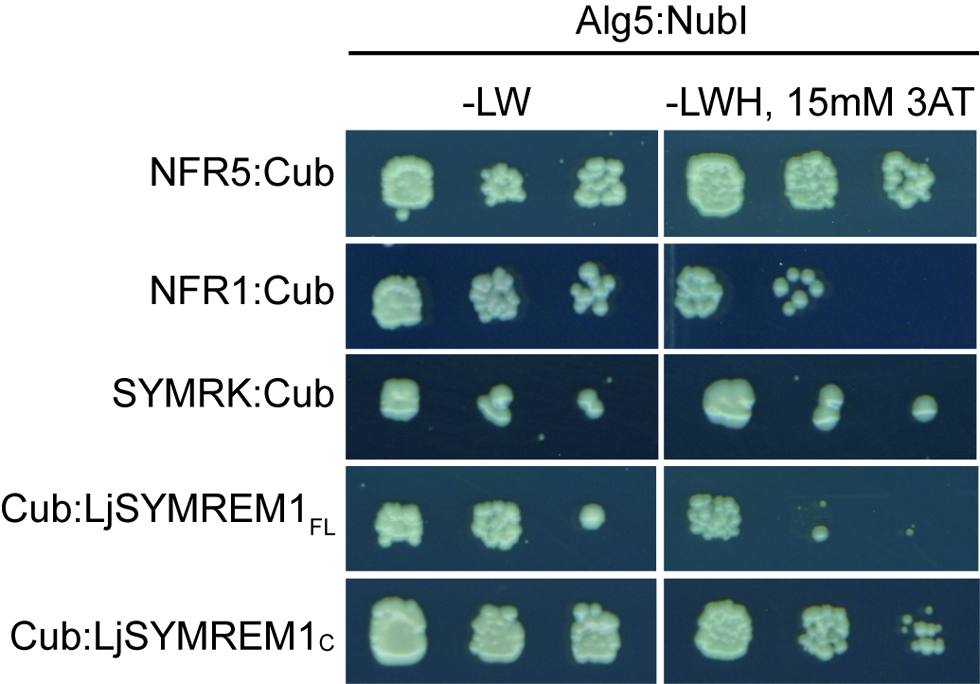

Supplement: Figure S3 — All membrane-anchored clones were expressed in the yeast split-ubiquitin system. The NubI tag is able to reconstitute together with Cub to the full-length ubiquitin and thus activates expression of the HIS3-reporter. Yeast growth on medium lacking leucine and tryptophan (−LW) shows the presence of both constructs. Interaction was tested on medium additionally lacking histidine (−LWH) that was supplemented with 15 mM 3-amino-1,2,4-triazole (3-AT) to suppress residual levels of endogenous histidine biosynthesis. (TIF) [file pone.0030817.s003.tif]

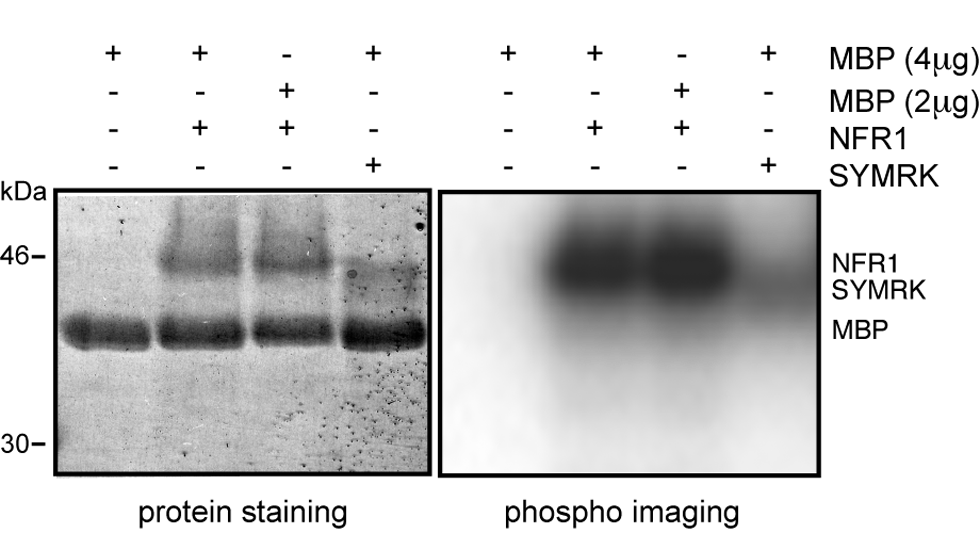

Supplement: Figure S4 — NFR1 and SYMRK kinase domains are unable to phosphorylate maltose binding protein (MBP). MBP was recombinantly expressed and purified. Since no phosphorylation of MBP was detected in kinase assays it can be concluded that phosphorylation that was observed with MBP-LjSYMREM1 does not derive from MBP phosphorylation. (TIF) [file pone.0030817.s004.tif]
