# Supplementary material for: Functional Domain Analysis of the Remorin Protein LjSYMREM1 in Lotus japonicus
Source: PLoS One. 2012 Jan 23;7(1):e30817. doi: 10.1371/journal.pone.0030817 (PMC3264624; doi:10.1371/journal.pone.0030817)
Supplement: Table S1 — Group 2 remorins exhibit unusual sequence diversity in their N-terminal region. Sequence comparison of full-length (overall), N- and C-terminal protein sequences of legume remorins that were found to be most closely related to each other (Figure 1) revealed that sequence conservation of the C-terminal region is in accordance with similarities of other legumes signaling proteins (Table S1B) while the N-terminal region is unusually diverse (Table S1A). (DOCX) [file pone.0030817.s005.docx]

**Sequence comparison between different group 2 Remorins that derived from the best BLAST hit against the genome sequences (Table S1A) and sequence analysis for symbiotic signaling proteins and nodulins (Table S1B).**

**Table S1A**

| **Identity/Similarity values in % for putative SYMREM1 homologs**  **(based on MtSYMREM1)** | | | | | | |
| --- | --- | --- | --- | --- | --- | --- |
| **domain** | ***Medicago*** | ***Lotus*** | **soybean** | **poplar** | **Common bean** | ***Vitis*** |
| **Full-length** | 100 | 55.5/67.1 | 53.1/65.1 | 49.8/62.9 | 52.4/65.0 | 42.9/59.0 |
| **C-term** | 100 | 72.9/85.3 | 67.4/79.1 | 63.6/76.0 | 67.4/79.1 | 58.9/76.0 |
| **N-term** | 100 | 27.2/38.3 | 30.0/42.5 | 26.3/40.8 | 27.3/41.6 | 15.8/30.3 |

*Medicago truncatula*= MtSYMREM1^#^; *Lotus japonicus*= chr4.CM0004.60.r2.d; soybean=Glyma08g01590.1; poplar= PtREM2.2*; common bean= Pv_TC37632; *Vitis vinifera*= Vv_XP_002267609; nomenclature introduced and sequences provided in Raffaele et al., (2007) (*) and Lefebvre et al. (2010) (#).

**Table S1B**

| ***Medicago*** | ***Lotus*** | **identity** | **similarity** |
| --- | --- | --- | --- |
| NFP | NFR5 | 72.0% | 82.8% |
| DMI2 | SYMRK | 81.6% | 87.6% |
| DMI1 | POLLUX | 80.8% | 85.3% |
| DMI3 | CCAMK | 85.7% | 92.2% |
| IPD3 | CYCLOPS | 78.2% | 87.1% |
| NIN | NIN | 57.4% | 67.5% |
| NSP2 | NSP2 | 73.9% | 83.4% |
| Leghemoglobin 1 | Leghemoglobin 1b | 70.3% | 80.4% |

Accession numbers for sequences used in this analysis: NFP (ABF50224), NFR5 (CAZ66917), DMI2 (CAD10811), SYMRK (AAM67418), DMI1 (AAS49490), POLLUX (BAD89022), DMI3 (Q6RET7), CCAMK (CAJ76700), IPD3 (ABN45743), CYCLOPS (ABU63668), MtNIN (ACN58567), LjNIN (CAB61243), MtNSP2 (CAH55768), LjNSP2 (BAE72690), Lb1 (P72992), Lb1b (BAB18108).
