# Supplementary material for: Functional Domain Analysis of the Remorin Protein LjSYMREM1 in Lotus japonicus
Source: PLoS One. 2012 Jan 23;7(1):e30817. doi: 10.1371/journal.pone.0030817 (PMC3264624; doi:10.1371/journal.pone.0030817)
Supplement: Table S2 — Testing interaction between LjSYMREM1 domains and NFR1 by FLIM analysis. LjSYMREM1:mOrange and NFR1:Cerulean were co-expressed in N. benthamiana leaves under control of the CaMV 35S-promoter. Shorter Cerulean lifetimes indicate interaction between the proteins. Strong interaction was observed between NFR1 and LjSYMREM1FL/LjSYMREM1C while mild but significant reduction in lifetime was also observed between NFR1 and LjSYMREM1N. Numeric values are provided in the table inset. Significance levels were calculated by student's t-test (with p<0.01 being significantly different). Free mOrange was co-expressed with NFR1:Cerulean to demonstrate that simple protein accumulation by over-expression of the acceptor fluorophore is not sufficient to reduce donor lifetimes. (PDF) [file pone.0030817.s006.pdf]

NFR1:Cerulean	+	+	+	+	+
free mOrange	-	+	-	-	-
SYMREM1:mOrange	-	-	$LjSYMREM1_{FL}$	$LjSYMREM1_{C}$	$LjSYMREM1_{N}$
lifetime [ns]	2.18	2.16	1.99	1.97	2.09
std. error	0.013	0.014	0.022	0.021	0.019
n	35	19	40	38	36
p-value	0.2221		$1.32 \times e^{-10}$	$1.47 \times e^{-10}$	0.0001
